# Reversal of Azole Resistance in *Candida albicans* by Human Neutrophil Peptide

**DOI:** 10.3390/biomedicines11020513

**Published:** 2023-02-10

**Authors:** Mohammad Imran Khan, Hani Choudhry, Sadaf Jahan, Irfan A. Rather

**Affiliations:** 1 Department of Biochemistry, Faculty of Science, King Abdulaziz University, Jeddah 21589, Saudi Arabia; 2 Centre of Artificial Intelligence for Precision Medicines, King Abdulaziz University, Jeddah 21589, Saudi Arabia; 3 Department of Medical Laboratory Sciences, College of Applied Medical Sciences, Majmaah University, Al-Majmaah 15341, Saudi Arabia; 4 Department of Biological Sciences, Faculty of Science, King Abdulaziz University, Jeddah 21589, Saudi Arabia; 5 Department of Applied Microbiology and Biotechnology, Yeungnam University, Gyeongsan-si 38541, Gyeongsanbuk-do, Republic of Korea

**Keywords:** *Candida albicans*, azole resistance, biofilms, human neutrophil peptide, combination therapy, drug efflux pumps

## Abstract

With the spread of AIDS and the increase in immunocompromised patients, multi-drug-resistant fungal infections have become a serious concern among clinicians, predominantly in the developing world. Therefore, developing novel strategies and new drugs is essential to overcome drug resistance in fungal pathogens. Antimicrobial peptides of human origin have been investigated as a potential treatment against Candida infections. In this study, human neutrophil peptide (HNP) was tested for its antifungal activity alone and in combination with fluconazole (FLC) against azole-susceptible and resistant *C. albicans* isolates, following CLSI guidelines. Susceptibility and combination interactions were also confirmed by MUSE cell viability assay and isobolograms for synergistic combinations, respectively. The effect of HNP on biofilm inhibition was determined spectrophotometrically and microscopically. Drug susceptibility testing showed minimum inhibitory concentrations (MICs) and minimum fungicidal concentrations (MFCs) ranging from 7.813 to 62.5 µg/mL and 15.625 to 250 µg/mL against all the tested *C. albicans* strains. The combination activity of FLC with HNP exhibited synergistic and additive interactions in 43% of each and indifferent interaction in 14%, and none of the combinations showed antagonistic interaction. Furthermore, HNB inhibited biofilm formation in all the tested *C. albicans* isolates. At the respective MICs, HNP exhibited inhibitory effects on the activity of the drug efflux pumps and their genes. These results warrant the application of HNP as a mono- or combination therapy with FLC to treat azole-resistant *C. albicans*.

## 1. Introduction

Candidiasis remains among the most frequent infections in cancer and HIV-infected patients, despite highly active antiretroviral therapy (HAART) [1,2]. Candidiasis caused by *Candida albicans* accounts for more than 30% of the world’s fungal mortality rates [3]. Three major classes of antifungal drugs are used for the management of candidiasis, which are polyenes (amphotericin B, nystatin, and natamycin), azoles (imidazoles and triazoles), and echinocandins (caspofungin, micafungin, and anidulafungin) [2]. Azoles, being the most widely used antifungal drug class, target fungal ergosterol biosynthesis pathway, and are also used as prophylaxis in patients in intensive care units or who have undergone invasive surgical procedures. The incidence of antifungal drug resistance in *C. albicans* has increased over the past decades [4]. Drug target alterations, biofilm formation, and reduced intracellular accumulation of the drugs are the most common mechanisms of drug resistance in *C. albicans* [5]. Mutation in the *ERG11* gene, which encodes for enzymes such as 14α-demethylase, is known to be associated with azole resistance in *C. albicans* [6,7]. Besides, there are two main classes of drug efflux pumps responsible for reducing intracellular drug accumulation in *C. albicans* cells. A number of studies have reported that azole-resistant isolates display transcriptional activation of ATP-binding cassette (ABC) and the major facilitator superfamily (MFS) genes encoding Cdr1p, Cdr2p, and MDR proteins [8,9].

Antimicrobial peptides have also been intensively studied for their antimicrobial and antibiofilm properties [5,10]. Combination therapy has also been studied as an alternative therapy to treat complex infectious diseases. It has been shown to have greater potential over monotherapy in alleviating the emergence of drug resistance and reducing dose-related toxicity, a major problem associated with azole drugs such as fluconazole. Previous studies have indicated that the combination of antifungal drugs with natural and synthetic compounds has the property to reverse azole drug resistance [11]. This study investigated the antifungal activity of HNP alone and in combination with fluconazole against *C. albicans* and their biofilm formation. The reversal of fluconazole resistance in *C. albicans* through the inhibition of the drug efflux pumps was also studied.

## 2. Materials and Methods

### 2.1. Fungal Strains

In this study, three fluconazole-susceptible and three fluconazole-resistant *Candida albicans* isolates, and a reference laboratory strain *C. albicans* SC5314 (ATCC), were used. All these strains were collected previously from different clinics and were stored in the department as glycerol stocks at −80 °C. Prior to the experiments, all the strains were revived on Sabouraud dextrose agar (SDA) and Sabouraud dextrose broth (SAB) purchased from Sigma Aldrich (St. Louis, MO, USA).

### 2.2. Antifungal Drug Susceptibility Testing

All seven isolates were initially screened for susceptibility against test peptide HNP (Thermo Fisher Scientific, Waltham, MA, USA) and fluconazole (FLC; Sigma-Aldrich) by broth microdilution method following CLSI recommended guidelines (CLSI, 2017). As a solvent, the stock concentrations tested for HNP and FLC were prepared to 1000 µg/mL using 1% dimethyl sulfoxide (DMSO; Sigma-Aldrich). In every set of experiments, culture (media and cells only) and negative (1% DMSO) controls were included. 

Each well without growth was subcultured onto agar plates and incubated at 37 °C for 24 h to determine the minimum fungicidal concentration (MFC). MFC was measured in the first well without any growth on the plate. For validation, experiments were conducted in triplicate. 

### 2.3. Combination Interactions and Isobolograms

The combination interaction of HNP with FLC was evaluated against the drug-resistant and susceptible *C. albicans* strains, as described previously [12]. HNP and FLC were combined in a 1:1 volume ratio to determine the fractional inhibitory concentration index (FICI), followed by nine varied ratio combinations (10:90, 20:80, 30:70, 40:60, 50:50, 60:40, 70:30, 80:20, 90:10). Isobolograms were constructed only for combinations which exhibited synergy in 1:1 combination ratios. Interactions were considered synergistic, additive, indifferent and antagonistic where FICI values were >0.5, 0.5–1, 1–4 and <4, respectively.

### 2.4. MUSE Viability Assay

The HNP was studied for its candidacidal property against *C. albicans* SC5314, FLC sensitive and FLC resistant, using MuseTM Count and Viability kit. The methodology was followed as suggested in the instruction manual. Briefly, *Candida* cells (5.0 × 10^6^ CFU/mL) were treated with HNP (MIC value), washed with PBS (Sigma-Aldrich, USA), and then mixed and incubated (37 °C for 5 min) with the reagents provided by the manufacturer. The samples were then analyzed by the MuseTM cell analyzer system. The obtained results were compared with the untreated cells (negative control).

### 2.5. Biofilm Assay

The impact of HNP on the inhibition of biofilm formation in *C. albicans* was evaluated as described previously [13]. The *C. albicans* strains, showing the highest MIC and MFC values, *C. albicans* SA42 (FLC sensitive), *C. albicans* SA04 (FLC resistant) and control strain SC5314 were undertaken for biofilm assay. Briefly, for the biofilm assay, 2 mL of freshly prepared inoculum (0.5 McFarland) was seeded in 12-well polystyrene plates. For biofilm formation, each plate was incubated at 37 °C for 90 min allowing the cells to adhere. After that, the wells were washed gently using PBS, followed by adding a sterile growth medium and incubating at 37 °C for 24 h. To investigate the inhibitory role of HNP on biofilm formation, the *C. albicans* cells were allowed to adhere to the polystyrene plate. Later, the biofilm was treated with various concentrations of HNP for 24 h at 37 °C. The fungal viability within the biofilms was also determined via an XTT (2,3-Bis-(2-Methoxy-4-Nitro-5-Sulfophenyl)-2*H*-Tetrazolium-5-Carboxanilide; Sigma-Aldrich, USA) reduction assay as described elsewhere [14]. The percentage inhibition of biofilm was calculated by using the following equation:

% Biofilm inhibition=[(control OD490 nm− test OD490 nm)/control OD490 nm]×100

Furthermore, the anti-biofilm potency of HNP was further validated with the help of Confocal laser scanning microscopy (CLSM) as described by Sun and co-workers. Briefly, the biofilms were grown on glass coverslips in a 24-well microtiter plate under standard conditions. After the adherence stage, the planktonic cells were washed out, fresh media supplemented with HNP (MIC value) was added to the designated wells, and plates were incubated for 24 h at 37 °C. Later, the coverslips were washed, fixed and stained with fluorescent dye concanavalin A (ConA)-Alexa Fluor 488 conjugate (Invitrogen Thermo Fisher Scientific; excitation wavelength = 488 nm and emission wavelength = 505 nm). Laser scanning confocal microscopy (Carl Zeiss, Inc., Oberkochen, Germany) was used to examine the slides.

### 2.6. Efflux Pumps Inhibitory Activity 

The effect of HNP on efflux pump inhibition was determined spectrophotometrically using rhodamine 6G (R6G; Sigma-Aldrich) assay following the previously described protocol [11]. Briefly, one FLC resistant (*C. albicans* SA04) and one FLC susceptible (*C. albicans* SA42) strain were grown up to the mid-log phase, followed by harvesting of cells by centrifuging at 4500× *g* for 2 min. The collected cell pellets were weighed, and 2 g of cells were suspended in 100 mL of PBS for all experiments. Prior to the experiment, cells were de-energised by growing cells for 1 h with 5 mM 2, 4-dinitrophenol (Sigma-Aldrich), and 2-deoxy-D-glucose (Sigma-Aldrich) at 37 °C. Post-incubation cells were collected, washed with PBS and exposed to HNP at their previously determined MIC values for 30 min. After exposure, 10 µM of R6G dye was added into the cell suspension, followed by incubation at 37 °C for 45 min. After incubation, all the cell suspensions were washed twice and re-suspended in PBS, followed by aliquoting 5 mL samples from each test at 5 min intervals for 25 min. For energy-dependent efflux activation, 0.1 M of glucose (Sigma-Aldrich) was added to the cell suspensions, followed by the withdrawal of 5 mL aliquots for up to 60 min. All the aliquots were centrifuged at 9000× *g* for 2 min, and the absorbances of supernatants were measured spectrophotometrically at 527 nm (SpectraMax iD3 multi-mode microplate reader, Molecular Devices, San Johe, CA, USA).

### 2.7. Cytotoxicity Studies

Cytotoxicity assay was performed following the previously described method [15]. Briefly, 5 mL of horse blood was centrifuged at 500× *g* for 5 min, and the supernatant containing blood plasma, leukocytes and platelets was discarded. The remaining pellet consisted of RBCs and was washed three times with 150 mM NaCl solution and centrifuged at 500× *g* for 5 min. A 2% erythrocyte cell suspension was prepared by adding 100 mL of sterile PBS (pH 7.4) to the cell pellet. The RBC suspension (180 µL) was added to tubes containing 20 µL of HNP in graded concentrations (7.81 µg/mL, 15.62 µg/mL, 31.25 µg/mL, 62.5 µg/mL, 125 µg/mL and 250 µg/mL), followed by 45 min incubation at 37 °C. All the tubes were cooled down and centrifuged at 1300× *g* for 5 min. A 100 µL of the supernatant was then diluted in 900 µL PBS. From each dilution, 200 µL was transferred to a 96-microtiter plate, and absorbance was recorded at 540 nm (SpectraMax iD3 multi-mode microplate reader, Molecular Devices). Two controls were prepared, negative control, which contained erythrocyte cell suspension and PBS, while the positive control consisted of erythrocyte cell suspension treated with 0.1% Triton X-100. Percentage hemolysis for each sample was calculated as follows:

$$\mathrm{Percentage}\;\mathrm{hemolysis}=\frac{\left[\mathrm{Absorbance}\;\mathrm{of}\;\mathrm{RBC}\;\mathrm{treated}\;\mathrm{with}\;\mathrm{test}\;\mathrm{compounds}-\mathrm{Absorbance}\;\mathrm{of}\;\mathrm{blank}\right]}{\left[\mathrm{Absorbance}\;\mathrm{of}\;\mathrm{RBC}\;\mathrm{treated}\;\mathrm{with}\;0.1\%\;\mathrm{tritonx}-100-\mathrm{Absorbance}\;\mathrm{of}\;\mathrm{blank}\right]}\times 100$$

### 2.8. Statistical Analysis

The obtained data were analyzed using Graph Pad Prism version 9.1.0 using student-*t* test (*p* value < 0.05). The results were represented as the average of three independent experiments (mean ± SD).

## 3. Results

### 3.1. Antifungal Susceptibility

The antifungal activity of HNP against drug-resistant and drug-susceptible *C. albicans* strains was determined by calculating MIC and MFC values, following CLSI-recommended guidelines [16]. HNP exhibited antifungal activity at varying levels against drug-resistant and susceptible *C. albicans* strains (Table 1). The MICs obtained for HNP ranged from 7.813 to 62.5 µg/mL, and these figures for FLC ranged from 0.5 to 250 µg/mL. 

The fungicidal activity of HNP was also investigated by determining MFC values against the test pathogens. All the MFCs of the HNP against both drug-resistant and susceptible strains are represented in Table 1. MFCs of HNP ranged between 15.625 and 250 µg/mL.

### 3.2. Combinations of HNP with FLC

#### Combinations in 1:1 Ratio

The antifungal activity of HNP in combination with the antifungal drug was investigated in a 1:1 ratio against both drug-resistant and susceptible strains. Based on FICI, most of the combinations between HNP and FLC against FLC-resistant strains exhibited a synergistic effect (Table 2). The combination activity of HNP and FLC against FLC susceptible isolates resulted in 50% indifferent effects, 25% additive, and 25% synergistic effects (Table 2). A total of 86% synergy and additive interactions were observed, with most of the synergistic interactions being against resistant isolates. Interestingly, no antagonistic drug interactions were observed following the combination of FLC with HNP against tested FLC drug-resistant and susceptible strains. 

### 3.3. Combinations in Varied Ratios

**Figure 1:**
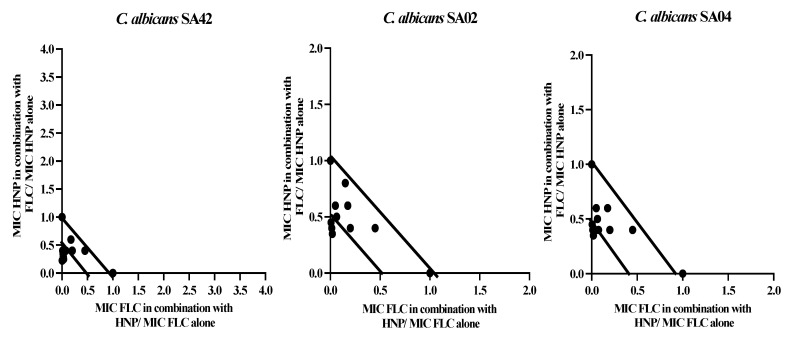
Isobolograms. The figure represents the synergistic and additive effects of nine different ratio combinations between human neutrophile peptide and fluconazole against fluconazole-susceptible *C. albicans* SA42 and fluconazole-resistant *C. albicans* SA02 and SA04.

All the synergistic interactions observed in the 1:1 ratio combination were further confirmed by nine different ratios between HNP and FLC, and the results are illustrated as isobolograms (Figure 1). The combination of HNP and FLC in nine ratios exhibited a strong synergistic effect in six ratios, and only three exhibited an additive effect in FLC-sensitive strain. HNP, in combination with FLC, showed three and four synergistic interactions and six and five additive interactions against two FLC-resistant strains (Figure 1). 

### 3.4. MUSE Viability Testing

**Figure 2:**
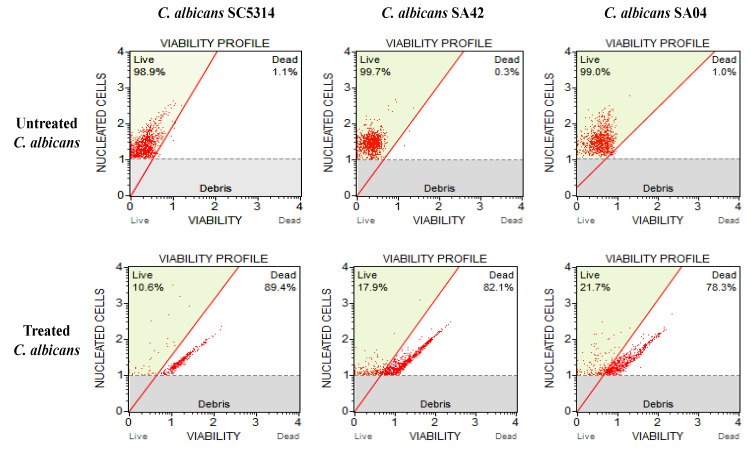
Effect of HNP on *C. albicans* cell viability. The figure shows *C. albicans* viability profile. Untreated control: unexposed *C. albicans* cells; *C. albicans* exposed to varied MIC values of the test compound.

The candidicidal activity of HPN at its MIC value was tested against *C. albicans*. The *C. albicans* strains, SA42 (FLC sensitive), SA04 (FLC resistant) and control strain SC5314 were used for the analysis as they showed the highest MIC and MFC values against HPN. The results demonstrated that HPN at its MIC value drastically affected the persistence of all the strains of *C. albicans* (Figure 2). HNP at 15.62 µg/mL (MIC for SA42) resulted in 82.1% dead cells whereas, at 62.5 µg/mL (MIC for SA04), it killed 78.3% *C. albicans* cells; its effect was most prominent in *C. albicans* SC5314, where 89.4% cells were found dead after treatment with HNP (7.81 µg/mL). Therefore, the viability results promote the candidacidal nature of HNP against FLC-susceptible and resistant *C. albicans*; however, further investigation on the mode of action of HNP is still needed to validate our results.

### 3.5. Biofilm Inhibition Property of HNP

**Figure 3:**
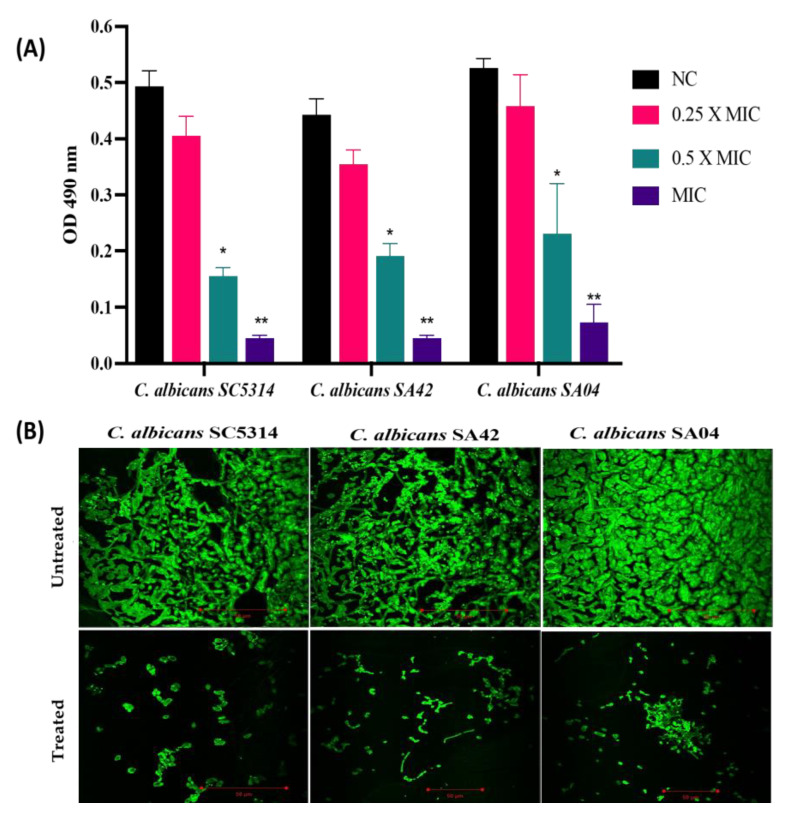
Antibiofilm activity of HNP against *C. albicans* biofilms. The figure shows the anti-biofilm potency of HNP against different strains of *C. albicans*, SA42 (FLC sensitive); SA04 (FLC resistant) and control strain SC5314. (**A**) The XTT assay was used to calculate the metabolic activity of cells embedded in biofilms and readings were recorded at 490 nm. NC, negative control. Statistical difference between test concentrations relative to the negative control was evaluated using Student’s unpaired two-tailed *t*-tests (** *p* value = 0.004; * *p* value = 0.02). (**B**) CLSM micrograph of biofilm formation in different strains of *C. albicans* where the biofilm matrix fluoresces green in colour due to presence of fluorescent dye Con A.

The impact of HNP on biofilm development was demonstrated by the metabolic activity of cells embedded in the biofilms (Figure 3A). The HNP abrogated the biofilm formation in all the *C. albicans* strains, and the results obtained from the XTT assay advocated the anti-biofilm property of HNP. HNP at 7.8 µg/mL resulted in an average biofilm inhibition of 90% in *C. albicans* SC5314, at 15.6 µg/mL abrogated the biofilm formation by an average percentage of 89.86% in *C. albicans* SA42 (FLC sensitive) whereas, at a concentration of 62.5 µg/mL, the average biofilm inhibition was 86.39% in *C. albicans* SA04 (FLC resistant). The obtained data suggested that HNP had maximum impact on *C. albicans* SC5314 followed by the *C. albicans* sensitive and then resistant strain; however, the test peptide successfully inhibited the biofilm in all tested pathogens and therefore, adds its potential to be a perfect anti-biofilm agent because it will minimize the chance of development of biofilm associated drug resistance in *C. albicans*.

The images obtained from CLSM further validated the in vitro XTT assay. The biofilm formed by untreated *C. albicans* was a dense three-dimensional mesh composed of yeast and hyphal structures. Since Con A has the ability to bind to the biofilm matrix, thus, the biofilm architecture was well-defined with bright green fluorescence. Among all the tested strains, *C. albicans* SC5314 developed the densest biofilms. However, the micrographs proved that biofilm formation was abrogated by HNP treatment (MIC value) in *C. albicans* strains. The biofilm in all the treated *C. albicans* strains was devoid of true hyphae and was composed of pseudo-hyphae and yeast cells. (Figure 3B).

### 3.6. Efflux Pumps Inhibitory Activity of HNP

**Figure 4:**
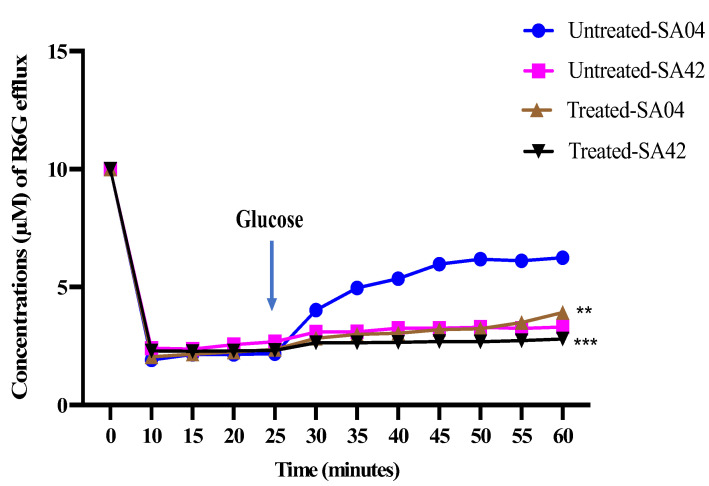
Effect of HNP on the efflux activity of different *C. albicans* strains. The figure represents the impact of HNP against efflux pumps in FLC-sensitive (*C. albicans* SA42) and resistant (*C. albicans* SA04) strains of *C. albicans*. The statistical difference between test was evaluated relative to the negative control using Student’s unpaired two-tailed *t*-tests (** *p* value = 0.005; *** *p* value = 0.0001).

The effect of HNP on the activity of the drug efflux pumps in FLC-resistant and susceptible *C. albicans* strains was investigated using R6G dye. In the FLC-susceptible strain (*C. albicans* SA42), the efflux of R6G dye in untreated glucose-starved cells ranged from 0.23 to 0.27 µM. The addition of glucose to the cell suspension slightly increased the efflux of R6G dye to 1.39 µM. HNP showed no effect in inhibiting the activity of the drug efflux pumps at previously determined MIC values, as no significant reduction was observed in the efflux of R6G dye in cells exposed to HNP compared to the untreated cells. In FLC-resistant strains (*C. albicans* SA04), the efflux of the dye in untreated glucose-starved cells ranged from 0.24 to 0.27 µM; however, the addition of glucose to the cell suspension stimulated the activity of the drug efflux pumps, and the amount of the dye effluxed from the cells increased from 2.18 to 4.02 µM in the first 5 min and to a maximum of 6.24 µM. HNP at MIC value was shown to be effective in reducing the activity of the drug efflux pumps. There was an increased inhibitory activity of the drug efflux pump even after 30 min when glucose was added, as is evident from Figure 4.

### 3.7. Cytotoxicity Effects 

**Figure 5:**
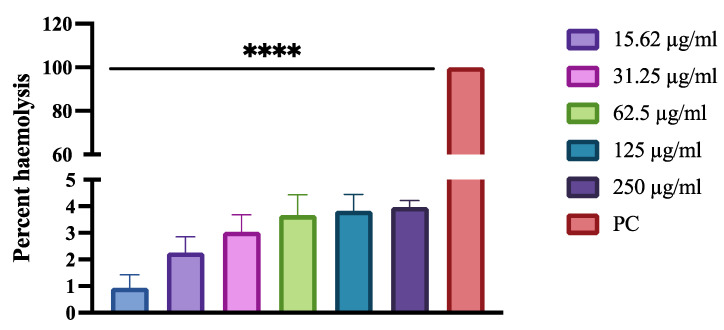
Percentage haemolysis of red blood cells following treatment with various concentrations of human neutrophile peptide. Hemolysis was calculated by an absorbance reading at 450 nm and compared to hemolysis achieved with 1% Triton X-100 (reference for 100% hemolysis). Bars with asterisks indicate significant differences as follows (**** *p* < 0.001).

The hemolytic effect of HNP was performed on horse red blood cells (RBC) to study their toxicity effect. HNP was tested in five different concentrations (7.81 µg/mL, 15.62 µg/mL, 31.25 µg/mL, 62.5 µg/mL, 125 µg/mL, and 250 µg/mL), which included the concentrations higher than previously determined MIC values against *C. albicans*. Incubation of the horse RBC with triton X-100 induced hemolysis of the cells resulting in the release of the cells constituents into the solution. However, an intact cell pellet in cells treated with PBS indicated that the RBC was not lysed. According to the obtained results, there was much less hemolysis in HNB-treated RBCs; at a high concentration of 250 µg/mL, a percent hemolysis of 3.96% was observed (Figure 5). 

## 4. Discussion

Candidiasis is a major health problem in immunocompromised individuals. Due to limited classes of antifungal drugs and their overuse in clinics, drug resistance in *C. albicans* has drastically increased [17,18,19,20]. Among all the antifungal drugs, FLC is the most widely used antifungal drug; therefore, resistance against FLC is widespread. Apart from the overuse of FLC in clinics, another factor contributing to the increased prevalence of FLC drug resistance is its fungistatic property. 

HNP is secreted by polymorphonuclear neutrophil granules and have been reported to have microbial-killing properties [21]. In this study, HNP exhibited potent antifungal activity against the tested *C. albicans* isolates. The MICs obtained for HNP against FLC-susceptible isolates were 7.813 and 15.625 µg/mL, whereas these figures against FLC resistant isolates ranged from 15.625 to 62.5 µg/mL. The MICs obtained for FLC against clinical isolates were 0.5 µg/mL, and for FLC-resistant isolates, were 125 and 250 µg/mL. According to the MIC breakpoints for FLC presented in the CLSI M27-S4E guidelines (MIC ≤ 8 μg/mL, susceptible; 16–32 μg/mL, susceptible-dose dependence; MIC ≥ 64 μg/mL, resistance), the test strains were categorized as susceptible and resistant to FLC. The antifungal activity of HNP slightly varies between FLC-susceptible and FLC-resistant *C. albicans* isolates. In the previous study, human neutrophil α-defensin 4 and its modified forms have reported similar antibacterial activity against drug-susceptible and drug-resistant bacteria [22]. HNP, a member of α-defensins, belongs to a family of small cationic antimicrobial peptides and is known to play an important role in innate immunity for host defense [21,22]. Besides anticandidal activity, α-defensins have been reported to inhibit the growth of *A. fumigatus* and *C. neoformans*. Therefore, the results obtained in this study and previous findings confirmed that HNPs have a potential antifungal property against different fungal pathogens.

The fractional inhibitory concentration index revealed that most of the combinations between HNP and FLC in a 1:1 ratio exhibited synergistic effects against FLC-resistant strains. This indicated that the antifungal activity of HNP with FLC is greater than the sum of the antifungal activity of the drugs used individually. In the study of Rather et al. [5], the combination activity of Human Cathelicidin LL-37 revealed 70% synergy with FLC and 100% synergy with amphotericin B and caspofungin. Our results are in agreement with these findings where we reported high synergistic interaction between HNP and FLC. The constructed isobolograms on synergistic interactions further supported the combination of HNP with FLC to treat resistant fungal pathogens. 

The main concern is that biofilm-associated infections display a higher level of antifungal drug resistance, and only few therapeutic agents can be employed to solve this problem [23,24,25]. Mostly, the antifungal drugs that inhibit *Candida* biofilms work on the initial phase of biofilm formation, thus impeding the cellular attachment to the substratum and subsequently, Ramagecompromising biofilm formation [26]. In the present study, HPN was capable enough to abrogate biofilm formation in both azole-sensitive and resistant strains of *C. albicans*. Previous studies have proved the anti-biofilm activity of antimicrobial peptides, Ctn, which demonstrated anti-biofilm activity and resulted in the compromised fungal plasma membrane in *C. albicans* [27]. Similarly, human α-defensin 6 (HD6) has been reported to block the adhesion of *C. albicans* epithelial cells, thereby preventing biofilm formation [28]. In tune with this, researchers have worked rigorously on various antimicrobial peptides to discover novel therapeutics against *Candida* infection and to prevent and treat biofilm-associated infections in immunocompromised patients [29,30]. 

Strong antifungal activity and greater synergistic interactions led this study to investigate the mechanism of drug reversal of FLC-resistant *C. albicans* isolates. Efflux pumps belonging to ABC transporters played a crucial role in developing azole drug resistance in *C. albicans.* Therefore, the effect of HNP on the efflux activity of these energy dependent pumps have been studied. Statistical analysis indicated no significant difference in the amount of the R6G pumped from FLC-resistant and susceptible cells in glucose-starved cells. Similar observations were reported by Khan et al. [31] and Gbelska et al. [32]. However, the addition of glucose to the cell suspension increased the efflux of the dye in the FLC-resistant strain. Glucose stimulated the activity of the drug efflux pumps, and there was remarkable difference observed in the efflux of the R6G dye before and after glucose was added to the cell suspension. 

The efflux of the dye in FLC-susceptible cells treated with HNP was all mostly the same as the efflux of the dye in untreated FLC-susceptible cells. This indicates no overexpression of the drug efflux pumps in susceptible strains. In resistant strains, decreased efflux of the dye was observed in cells treated with HNP as compared to untreated resistant cells. Yaojun et al. [33] indicated that *C. albicans* strains with knocked-out CDR1 and CDR2 genes showed a decreased efflux of R6G dye in multidrug-resistant strains. This proves that cdr1 and cdr2 are responsible proteins for conferring FLC drug resistance in *C. albicans* strains. Our results are congruent with these findings, where higher efflux in resistant isolates of *C. albicans* can be related to the overexpression of the efflux pumps. HNP in this study has shown the efflux pump inhibitory activity and thereby, chemosensitizer-resistant strains to azole drugs. In the previous study, synthetic peptides and octapeptides have been reported to reverse the azole resistance in FLC-resistant isolates [34]. Further studies will be required to understand whether HNP is very specific and only inhibits a narrow range of efflux pumps or has broader application to inhibit both ABC and MFS transporters. 

HNP, besides being reported to have antimicrobial activity, is also known to modulate immune responses [35]. Further studies in this area will be beneficial to understand the synergistic interaction of these approaches of killing fungal pathogens and enhancing the host immune responses. Such studies will be beneficial as patients with compromised immunity are more prone to fungal infections. The dual benefit of such peptides can be utilized to develop novel antifungal drugs, showing efficient results in immunocompromised patients.

## 5. Conclusions

*Candida albicans* is the most problematic *Candida* species responsible for most *Candida*-related infections. A very high percentages of drug resistance to FLC and other antifungal drugs were reported in *C. albicans*. HNP possesses antifungal and antibiofilm activities against *C. albicans* at acceptable concentrations and can be used as a potential treatment for *C. albicans* infections. The synergistic effect of NHP in combination with FLC might be a promising strategy to combat drug-resistant *C. albicans* infections. This study also revealed that HNP could inhibit efflux pumps and has chemosensitizing potential with FLC against resistant pathogens. High antifungal activity, synergistic interaction with known drugs, and low toxicity of HNP demand further studies to unveil the clinical relevance of this peptide for the antifungal treatment of *Candida* infections.

## Figures and Tables

**Table 1:** Antifungal activity of human neutrophil B peptide against fluconazole susceptible and fluconazole resistant *Candida albicans* isolates.

Isolates		HNB		FLC
		MIC (µg/mL)	MFC (µg/mL)	MIC (µg/mL)
FLC susceptible	SC5314	7.813	15.625	0.5
	SA91	7.813	15.625	0.5
	SA19	7.813	31.25	0.5
	SA42	15.625	31.25	0.5
FLC resistant	SA02	31.25	62.5	125
	SA61	15.625	62.5	125
	SA04	62.5	250	250

**Table 2:** Antifungal activity of human neutrophil B peptide in combination with fluconazole in a 1:1 ratio against fluconazole susceptible and fluconazole resistant *Candida albicans* isolates.

*Candida* Isolates		FICA	FICB	ΣFIC	INT
FLC susceptible	SC5314	0.250	0.500	0.750	ADD
	SA91	0.125	0.500	0.625	ADD
	SA19	1.000	0.063	1.063	IND
	SA42	0.031	0.250	0.281	SYN
FLC resistant	SA02	0.250	0.250	0.500	SYN
	SA61	0.500	0.063	0.563	ADD
	SA04	0.031	0.125	0.156	SYN

## Data Availability

The data available is cited in the text.
